# Supplementary figures and images for: Personalized education approach based on cognitive psychology for endoscopic diagnosis: A multicenter randomized trial
Source: PLoS One. 2025 Sep 17;20(9):e0332708. doi: 10.1371/journal.pone.0332708 (PMC12443239; doi:10.1371/journal.pone.0332708)

# Supplementary Figure 1

(A)

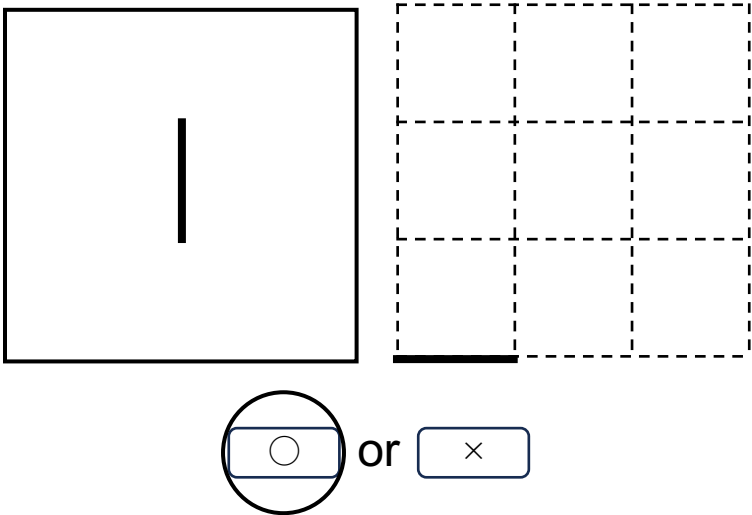

(B)

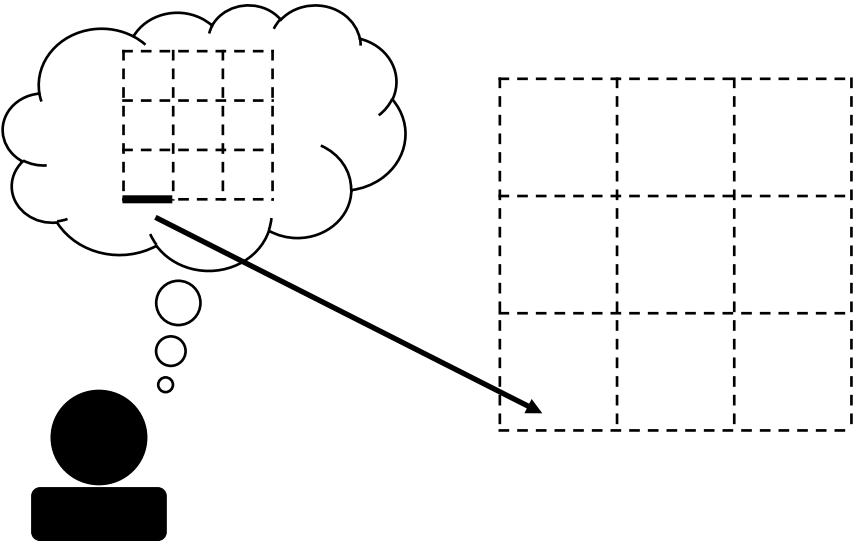

Supplement: S1 Fig — (A) First, respondents memorized the line length shown on the left. Subsequently, they answered whether the line on the right that appears is the same length as the line they memorized. (B) They pointed to the line’s location memorized in (A) in the 3 × 3 square where the line is not drawn. These (A) and (B) were first presented one question at a time, then two, then three, and so on, increasing the number and location of lines to be memorized. (PDF) [file pone.0332708.s001.pdf]

Supplementary Figure 2

(A)

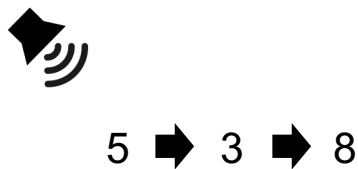

(B)

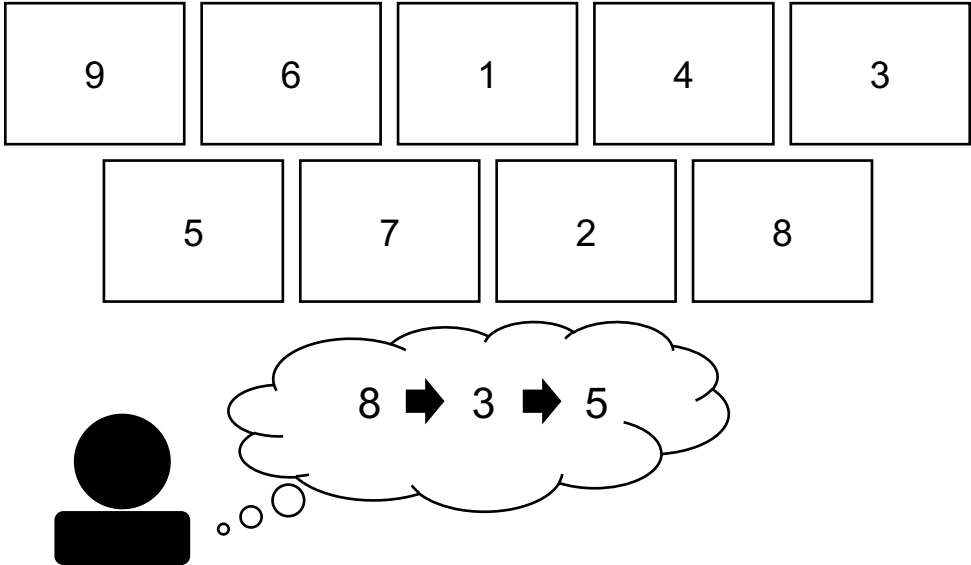

Supplement: S2 Fig — (A) Respondents heard numbers from 1 to 9 through audio and memorized them. (B) They then selected the numbers from a list of candidates in reverse order. The numbers were presented in random order, as shown in the figure. Each time respondents answered a question correctly, the number of numbers presented increased. (PDF) [file pone.0332708.s002.pdf]

# Supplementary Figure 3

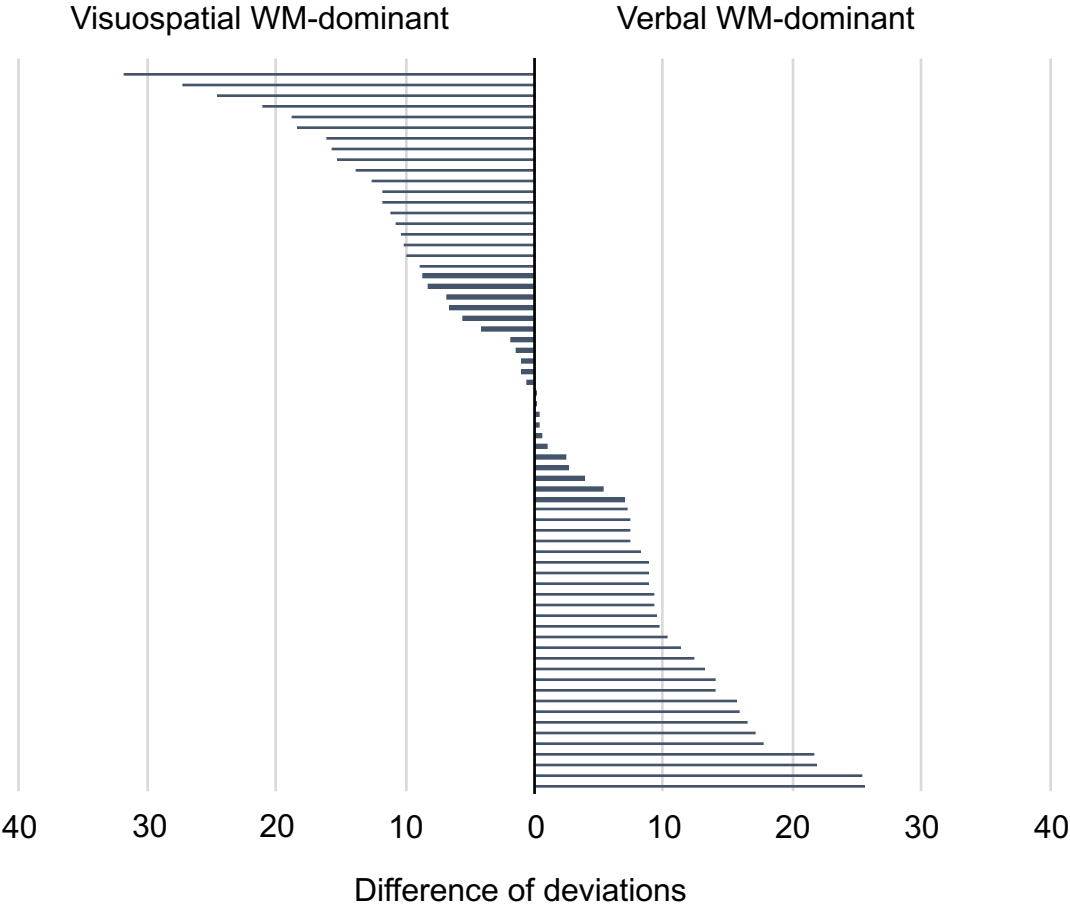

Supplement: S3 Fig — The difference between the deviations of the visuospatial and verbal WM was displayed as a bar graph for each endoscopist participating in the development phase. The visuospatial WM is dominant if the bars are shown in the left direction, and the verbal WM is dominant if the bars are displayed in the right direction. (PDF) [file pone.0332708.s003.pdf]
