# Supplementary material for: Personalized education approach based on cognitive psychology for endoscopic diagnosis: A multicenter randomized trial
Source: PLoS One. 2025 Sep 17;20(9):e0332708. doi: 10.1371/journal.pone.0332708 (PMC12443239; doi:10.1371/journal.pone.0332708)
Supplement: S1 Table — (DOCX) [file pone.0332708.s004.docx]

**S1 Table. NICE classification**

|  | Type 1 | Type 2 | Type 3 |
| --- | --- | --- | --- |
| Color | Same or lighter than background mucosa | Browner compared to background mucosa | Brown to dark brown compared to background mucosa; sometimes patchy white areas |
| Vessels | None, or isolated lacy vessels may be present across the lesion. | Brown vessels surrounding white structures | Area of disrupted or missing vessels |
| Surface pattern | Dark or white spots of uniform size, or homogeneous pattern | Oval, tubular, or branched white structures surrounded by brown vessels | Amorphous or absent surface pattern |
| Most likely pathology | Non-neoplastic lesion (hyperplastic polyp) or sessile serrated lesion | Adenoma or intramucosal cancer* | Invasive cancer |

*Type 2 consists of the Vienna classification types 3, 4, and superficial 5 (all adenomas with either low- or high-grade dysplasia or superficial submucosal carcinoma). High-grade dysplasia or superficial submucosal carcinoma may be suggested by an irregular vessel or surface pattern and is often associated with an atypical morphology.
