# Supplementary material for: Personalized education approach based on cognitive psychology for endoscopic diagnosis: A multicenter randomized trial
Source: PLoS One. 2025 Sep 17;20(9):e0332708. doi: 10.1371/journal.pone.0332708 (PMC12443239; doi:10.1371/journal.pone.0332708)
Supplement: S2 Table — (DOCX) [file pone.0332708.s005.docx]

**S2 Table. Baseline characteristics of endoscopists for the development of standard working memory profile**

|  |  |
| --- | --- |
| Age (years, mean ± SD) | 41.0 ± 7.6 |
| Sex (male / female) | 69 / 10 |
| Experience of colonoscopies |  |
| < 100 | 5 |
| < 500 | 15 |
| < 5000 | 27 |
| ≥ 5000 | 32 |
| Certification as specialist (yes / no) | 53 / 26 |
| Number of colonoscopies per year |  |
| < 100 | 23 |
| < 500 | 39 |
| < 5000 | 17 |

Specialists were endoscopists certified by the Japan Gastroenterological Endoscopy Society. Age is expressed as mean ± standard deviation.
