# Supplementary material for: Personalized education approach based on cognitive psychology for endoscopic diagnosis: A multicenter randomized trial
Source: PLoS One. 2025 Sep 17;20(9):e0332708. doi: 10.1371/journal.pone.0332708 (PMC12443239; doi:10.1371/journal.pone.0332708)
Supplement: S3 Table — (DOCX) [file pone.0332708.s006.docx]

**S3 Table. Diagnostic accuracy according to the training program in the Matched-E and Unmatched-E groups.**

|  | Visuospatial education program | Verbal education program | P-value |
| --- | --- | --- | --- |
| Matched-E group |  |  |  |
| Non-neoplastic lesion | 69.3% | 63.8% | P= 0.540 |
| Adenoma/intramucosal cancer | 61.4% | 45.5% | P= 0.016 |
| Invasive cancer | 58.1% | 57.1% | P= 0.927 |
| Unmatched-E group |  |  |  |
| Non-neoplastic lesion | 47.6% | 53.2% | P= 0.921 |
| Adenoma/intramucosal cancer | 53.6% | 49.8% | P= 0.183 |
| Invasive cancer | 56.7% | 52.3% | P= 0.421 |
