# Supplementary material for: Personalized education approach based on cognitive psychology for endoscopic diagnosis: A multicenter randomized trial
Source: PLoS One. 2025 Sep 17;20(9):e0332708. doi: 10.1371/journal.pone.0332708 (PMC12443239; doi:10.1371/journal.pone.0332708)
Supplement: Attachment — Submitted filename: P-by-P_response_0807.docx [file pone.0332708.s008.docx]

August 7, 2025

Emily Chenette

Editor-in-Chief

*PLOS ONE*

Dear Editor:

On behalf of all the authors, I thank you for reviewing our manuscript titled “**Personalized education approach based on cognitive psychology for endoscopic diagnosis: a multicenter randomized trial.**”

We appreciate your valuable comments that have helped us to improve our manuscript substantially. We have revised the manuscript in line with your suggestions and have highlighted all related changes in red. Additionally, we have provided point-by-point responses to the reviewers’ comments attached below.

Thank you for your consideration. I look forward to hearing from you.

Sincerely,

Fumiaki Ishibashi

Department of Gastroenterology, International University of Health and Welfare Ichikawa Hospital

6-1-14, Konodai, Ichikawa-shi, Chiba 272-0827, Japan

Email: f.jazzmaster@gmail.com

Phone: +81-47-375-1111

Fax: +81-47-373-4921

***Editorial and Journal Office Requirements***

**Comment 1:**

Please ensure that your manuscript meets PLOS ONE's style requirements, including those for file naming.

**Response to Comment 1:**

We have revised our manuscript according to the PLOS ONE formatting style. File names have also been updated accordingly.

**Comment 2:**

Thank you for stating the following financial disclosure:

“This work was supported by International University of Health and Welfare Research Funds for Academic Year 2023.”

Please state what role the funders took in the study.  If the funders had no role, please state: "The funders had no role in study design, data collection and analysis, decision to publish, or preparation of the manuscript.” If this statement is not correct you must amend it as needed.

Please include this amended Role of Funder statement in your cover letter; we will change the online submission form on your behalf.

**Response to Comment 2:**

As the funders had no role in the study, we have added the following statement in the cover letter as requested: "The funders had no role in study design, data collection and analysis, decision to publish, or preparation of the manuscript."

**Comment 3:**

Funding information should not appear in the Acknowledgments section or elsewhere in the manuscript. We will only publish funding information present in the Funding Statement section of the online submission form. Please remove any funding-related text from the manuscript. 

**Response to Comment 3:**

As per the editorial office’s indication, funding information has been removed from the Acknowledgments and other sections of the manuscript.

**Comment 4:**

Thank you for stating the following in the Competing Interests section:

“FI received honoraria for lectures from the Fujifilm Corporation. ME is a consultant for AI Medical Service, Inc. CK received honoraria for lectures from the Fujifilm Corporation and Olympus Corporation. TG received honoraria for lectures from the Fujifilm Corporation, Fujifilm Medical, and Olympus Corporation. SS received honoraria for lectures from the Fujifilm Corporation and Olympus Corporation. The authors declare no conflicts of interest.”

Please confirm that this does not alter your adherence to all PLOS ONE policies on sharing data and materials, by including the following statement: "This does not alter our adherence to PLOS ONE policies on sharing data and materials.” (as detailed online in our guide for authors <http://journals.plos.org/plosone/s/competing-interests>).  If there are restrictions on sharing of data and/or materials, please state these. Please note that we cannot proceed with consideration of your article until this information has been declared.

Please include your updated Competing Interests statement in your cover letter; we will change the online submission form on your behalf.

**Response to Comment 4:**

We have revised the Competing Interests section in the cover letter to include the following statement as per your suggestion: "This does not alter our adherence to PLOS ONE policies on sharing data and materials."

**Comment 5:**

We note that you have included the phrase “data not shown” in your manuscript. Unfortunately, this does not meet our data sharing requirements. PLOS does not permit references to inaccessible data. We require that authors provide all relevant data within the paper, Supporting Information files, or in an acceptable, public repository. Please add a citation to support this phrase or upload the data that corresponds with these findings to a stable repository (such as Figshare or Dryad) and provide and URLs, DOIs, or accession numbers that may be used to access these data. Or, if the data are not a core part of the research being presented in your study, we ask that you remove the phrase that refers to these data.

**Response to Comment 5:**

As the data were not a core part of the research being presented in this study, the related sentences were removed from the manuscript.

**Comment 6:**

Your ethics statement should only appear in the Methods section of your manuscript. If your ethics statement is written in any section besides the Methods, please move it to the Methods section and delete it from any other section. Please ensure that your ethics statement is included in your manuscript, as the ethics statement entered into the online submission form will not be published alongside your manuscript.

**Response to Comment 6:**

We have confirmed that the ethics statement appears only in the Methods section in the revised manuscript.

***Reviewer #1***

**Comment 1:**

The sample size was 60 trainees at the start of the study, but only 40 were ultimately analyzed, so there is a possibility that the statistical power is insufficient.

**Response to Comment 1:**

Thank you for your pertinent comment. As you have pointed out, the final analysis included 40 participants, which means 20 participants dropped out from the initial 60 registered at the time of enrollment. However, per our analysis, the minimum sample size required to assess the study endpoint was 34 participants. Therefore, 40 participants was deemed to be sufficient to determine the endpoint with a 10% difference in diagnostic accuracy (α = 0.05, power = 0.80). Moreover, based on the actual observed data (delta= 7.7%, standard deviation= 9.6%), the number of analyzed participants yielded adequate statistic power (94.3%). We have explained this in the Discussion section in the revised manuscript.

Page 21 Line 8–10 (Discussion): Furthermore, based on the actual observed data (delta= 7.7%, standard deviation= 9.6%), the number of analyzed participants yielded adequate statistic power (94.3%). This reinforces the validity of this study.

**Comment 2:** I am concerned about the large proportion of "dropouts" in the RCT and the fact that the reasons for dropouts were not explained in detail.

**Response to Comment 2:**

Thank you for your pertinent comment. The participants in this study were hospital residents who belonged to various departments, including those other than gastroenterology. Therefore, not all participants were fully motivated and committed to the study, despite their initial agreement to participate. Nevertheless, after excluding these dropouts, the final number of participants analyzed reached the same sample size as the preceding one. The detailed reasons for dropout have now been explained in the figure legend to maintain the transparency of the study flow (Fig 1. Study flow diagram). Additionally, an explanation has been included in the Discussion section.

Page 21, Line 3–8 (Discussion): Fifth, a substantial number of dropouts were observed in this study. The enrolled participants were hospital residents from various departments, including those outside of the gastroenterology department. Therefore, not all participants were entirely motivated and committed to the study, despite their initial agreement to participate. Nevertheless, the final number of participants analyzed reached the same sample size as the preceding one after excluding these dropouts.

**Comment 3:** The improvement in diagnostic accuracy for non-neoplastic lesions was significant (68.0% vs. 48.4%, P=0.002), and I agree that it may contribute to reducing unnecessary treatment.

On the other hand, there was no difference in diagnostic accuracy for neoplastic lesions (56.7% vs. 56.7%, P=0.998). What is the reason for this?

**Response to Comment #3:**

Thank you for your pertinent comment. The primary reason for the lack of difference in the diagnostic accuracy for neoplastic lesions is that non-neoplastic and neoplastic lesions exhibit distinct surface and vascular patterns, allowing trainees to easily acquire the skill of distinguishing these lesions even after brief education. In contrast, neoplastic lesions such as adenoma and cancer share similar surface and vascular patterns, making it challenging to distinguish between them. Therefore, a longer training intervention may be necessary to observe the difference in distinction accuracy for neoplastic lesions between the Matched-E and Unmatched-E groups. This explanation has been included in the revised Discussion section.

Page 18 Line 17– Page 19 Line 4 (Discussion): Trainees can easily acquire the skill of distinguishing non-neoplastic and neoplastic lesions even after brief education because these lesions exhibit distinct surface and vascular patterns. In contrast, among the neoplastic lesions, adenomas and cancer share similar surface and vascular patterns, making it challenging to distinguish between them. Thus, a longer training intervention may be necessary to observe the difference in distinction accuracy for neoplastic lesions between the Matched-E and Unmatched-E groups.

**Comment 4:** There has been only limited discussion about the applicability of endoscopic education using WM profiling to actual clinical practice, so please add more discussion.

**Response to Comment 4:**

We appreciate your valuable comment. Notably, our proof-of-concept study demonstrated the potential for tailoring endoscopic education according to individual WM profiles, particularly in the context of acquiring diagnostic skills for colorectal polyps through a brief, 10-min training session. The results revealed a statistically significant benefit in short-term educational effectiveness when trainees were provided with instructional methods aligned with their WM profile, broadly categorized as either verbal- or visuospatial-dominant. However, endoscopic training in real-world clinical settings is usually a combination of verbal and visuospatial modalities. Therefore, mutual understanding between trainees and trainers regarding the most suitable instructional approaches may enable the implementation of more efficient and focused training, even within the constraints of busy clinical environments. This discussion about the applicability of endoscopic education using WM profiling to actual clinical practice have been included in the Discussion section. Additionally, we have explained the possibility of clinical application of WM-based training beyond the colorectal polyp diagnosis, eg, ESD and ERCP in the following sentences.

Page 19 Line 5–11 (Discussion): However, in real-world clinical settings, endoscopic training is rarely limited to purely verbal or purely visuospatial modalities; rather, a combination of both is typically employed. Therefore, employing either description text-based learning or pattern recognition learning alone as a standalone educational method, as used in this study, would be of limited practical value. Consequently, fostering mutual understanding between trainees and trainers regarding the most suitable instructional approaches may enable the implementation of more efficient and focused training, even within the constraints of busy clinical environments.

**Comment 5:** Although a brief 10-minute education session has been shown to be effective, the long-term effects (e.g., maintaining diagnostic accuracy several months later) have not been evaluated.

**Response to Comment 5:**

Thank you for your insightful comment. In real-world educational settings, training rarely terminates with a single session; rather, it is a repetitive and ongoing process. Therefore, assessing whether a one-time educational intervention has a measurable impact several months later may be of clinical relevance. Nevertheless, we acknowledge this as a limitation of our study and have added the following sentences to the Discussion section.

Page 21 Line 10-12 (Discussion): Finally, the long-term effects of the pedagogy used in this study are undetermined. Consequently, a long-term prospective study is warranted to confirm the results of this study.

**Reviewer #2**

**Comment 1:**

Clinical relevance for neoplastic lesions: Crucially, your study found no significant difference in diagnostic accuracy for neoplastic lesions (adenoma/cancer) between the two educational groups. As an accurate diagnosis of these lesions is the primary goal of endoscopy, the lack of improvement in this area significantly limits the clinical utility of your personalised educational approach. While differences were observed for non-neoplastic lesions, their overall accuracy remained relatively low (68.0%).

**Response to Comment 1:**

Thank you for your pertinent comment. The primary endpoint of this study was the proportion of correct diagnoses for non-neoplastic lesions, adenomas/intramucosal cancers, and invasive cancers, representing the diagnostic accuracy for each pathological category. As a secondary endpoint, we evaluated the ability to distinguish each pathological category from the other two. The results showed that the ability to differentiate non-neoplastic lesions from the other two categories (adenomas/intramucosal cancers and invasive cancers) was significantly higher in the Matched-E group than that in the Unmatched-E group. This finding indicates the acquisition of clinically important diagnostic competence in distinguishing non-neoplastic from neoplastic lesions. We have added the following sentences in the Method section to clearly explain this aim. Additionally, we have discussed the differential acquisition of diagnostic skills between neoplastic and non-neoplastic lesions in the Discussion section.

Moreover, as this study was positioned as a proof-of-concept study to explore the feasibility of implementing tailored education based on trainees’ WM profiles, its primary significance lies in proposing a novel concept: the integration of cognitive psychology into endoscopic training. As an initial step toward proposing this concept, we evaluated outcomes that could be measured within a short period, using the minimum required sample size for a clinical study. Therefore, we believe that the lack of statistically significant improvement in the differential diagnosis of neoplastic lesions, and the relatively low diagnostic accuracy of 68.0% for non-neoplastic lesions should not be viewed as critical limitations. We have included a discussion of the study's proof-of-concept nature in the revised manuscript.

Page 11 Line 5–6 (Methods): Additionally, the diagnostic accuracy according to the lesion pathology was evaluated to determine the ability to distinguish each pathological category from the other two.

Page 16 Line 17– Page 17 Line 1 (Discussion): Specifically, this proof-of-concept study explored the feasibility of implementing tailored education based on trainees’ WM profiles. The study proposed a novel concept: integrating cognitive psychology into endoscopic training.

Page 18 Line 17– Page 19 Line 4 (Discussion): Trainees can easily acquire the skill of distinguishing non-neoplastic and neoplastic lesions even after brief education because these lesions exhibit distinct surface and vascular patterns. In contrast, among the neoplastic lesions, adenomas and cancer share similar surface and vascular patterns, making it challenging to distinguish between them. Thus, a longer training intervention may be necessary to observe the difference in distinction accuracy for neoplastic lesions between the Matched-E and Unmatched-E groups.

**Comment 2:**

Mismatch of Learning Approach with Task Nature: Endoscopic diagnosis, particularly when using systems such as the NICE classification, is a highly visual task that requires pattern recognition. Using a 'descriptive text'-based education approach for verbally dominant learners, while aligning with their working memory (WM) type, may not be the most effective way to teach a task that relies so heavily on visual cues. It is difficult to extrapolate how this methodology would generally enhance visual diagnostic abilities.

**Response to Comment 2:**

Thank you for your insightful comment. As you have pointed out, polyp diagnosis is a highly visual task. Furthermore, while endoscopic diagnostic education based on written descriptions may serve as a useful aid for verbal WM-dominant trainees, we also recognize that such an approach alone does not constitute a comprehensive educational method. Consequently, in actual educational settings, trainers rarely rely exclusively on either verbal instruction or visuospatial pattern recognition when teaching trainees; instead, a combination of both approaches is typically employed. Therefore, evaluating the superiority of one instructional method over the other for a specific diagnostic task may hold limited inherent value. However, what we wish to emphasize in this study is the importance of the trainees and trainers understanding of the trainee’s WM profile. This understanding may help them reach a shared perspective on which instructional methods should be emphasized in clinical practice.

Accordingly, in the revised manuscript, we emphasized that this study is a proof-of-concept study; therefore, further research should investigate the effectiveness of WM-tailored educational strategies that are more directly applicable to real-world clinical training. In addition, the following explanation has been added to the Discussion section.

Page 19 Line 5–11 (Discussion): However, in real-world clinical settings, endoscopic training is rarely limited to purely verbal or purely visuospatial modalities; rather, a combination of both is typically employed. Therefore, employing either description text-based learning or pattern recognition learning alone as a standalone educational method, as used in this study, would be of limited practical value. Consequently, fostering mutual understanding between trainees and trainers regarding the most suitable instructional approaches may enable the implementation of more efficient and focused training, even within the constraints of busy clinical environments.
