# Supplementary material for: Personalized education approach based on cognitive psychology for endoscopic diagnosis: A multicenter randomized trial
Source: PLoS One. 2025 Sep 17;20(9):e0332708. doi: 10.1371/journal.pone.0332708 (PMC12443239; doi:10.1371/journal.pone.0332708)
Supplement: Attachment — Submitted filename: P-by-P_response_0902.docx [file pone.0332708.s009.docx]

September 2, 2025

Emily Chenette

Editor-in-Chief

*PLOS ONE*

Dear Editor:

On behalf of all the authors, I would like to thank you for reviewing our manuscript titled “**Personalized education approach based on cognitive psychology for endoscopic diagnosis: a multicenter randomized trial.**”

We greatly appreciate your valuable comments, which have helped us substantially improve the manuscript. We have revised it according to your suggestions, with all related changes indicated in red. Additionally, we have provided detailed point-by-point responses to the reviewers’ comments, as attached below.

Thank you for your consideration. I look forward to hearing from you.

Sincerely,

Fumiaki Ishibashi

Department of Gastroenterology, International University of Health and Welfare Ichikawa Hospital

6-1-14, Konodai, Ichikawa-shi, Chiba 272-0827, Japan

Email: f.jazzmaster@gmail.com

Phone: +81-47-375-1111

Fax: +81-47-373-4921

**Reviewer #2**

**Comment 1:**

Recognition of a Major Limitation
The absence of improvement in the diagnosis of neoplastic lesions—particularly adenomas and cancers—should be explicitly recognized as a major limitation, even in the context of a proof-of-concept study. Minimizing the importance of this finding risks underrepresenting its impact on the clinical applicability of the proposed educational approach.

**Response to Comment 1:**

Thank you very much for this important comment. As the reviewer correctly pointed out, in our study, WM-based individualized education improved the ability to distinguish non-neoplastic from neoplastic lesions; however, it did not enhance diagnostic accuracy in differentiating adenomas from invasive cancers. We recognize this as a major limitation and agree that the educational approach used in this study cannot yet be directly applied to clinical practice.

In the revised manuscript, we have added a new paragraph to the Discussion section to explain these points, and we have modified the Conclusion to describe the implications of our findings in a more cautious manner.

Page 19, Lines 321-322 (Discussion): The lack of improvement in diagnostic accuracy for adenomas and cancers represents the most important limitation of this study for two main reasons.

Page 22, Lines 381-385 (Conclusion): However, the lack of improvement in diagnostic accuracy for neoplastic lesions after short-term training remains a notable limitation. In clinical practice, it is difficult to fully stratify trainees into verbal or visual WM groups and implement strictly modality-specific training. Addressing this limitation will be essential to achieve truly individualized education.

**Comment 2:**

Plausible Underlying Mechanism
In addition to morphological similarity between adenomas and early cancers, a plausible contributing factor may be a mismatch between the instructional modality and the inherently visual nature of the diagnostic task. A text-based, verbal WM–aligned approach may be insufficient for developing advanced visual pattern recognition skills. This possible mechanism should be incorporated into the Discussion to provide a more comprehensive interpretation of the findings.

**Response to Comment 2:**

We sincerely appreciate this important comment. We believe there are two main reasons why diagnostic accuracy for adenomas and cancers did not improve.

First, as noted in our previous revision, adenomas and cancers share morphological features such as surface structure, which makes it difficult to achieve meaningful learning effects through short-term training.

Second, as the reviewer rightly pointed out, the diagnosis of colorectal polyps inherently requires visual assessment. Therefore, when a text-based educational program was provided to participants with predominantly verbal WM, a mismatch may have occurred between the instructional modality and the visual nature of the diagnostic task. Indeed, even within the Matched-E group, diagnostic accuracy for adenomas and intramucosal cancers was lower in the verbal-education subgroup compared with the visual-education subgroup (45.5% vs. 61.3%, P= 0.016; Supplementary Table 3). This suggests that verbal instruction alone may not have been an appropriate method for fostering polyp diagnostic ability in this context. In particular, the second reason highlights why direct application of this educational approach to clinical practice is challenging. In real-world settings, it is not feasible to clearly stratify trainees into verbal- or visual-WM dominant groups and provide completely segregated training methods.

We have now explicitly acknowledged in the Discussion section that this constitutes the major limitation of our study. In addition, we have clarified that in practical educational settings, even for trainees with predominantly verbal WM, an approach relying exclusively on verbal instruction would be unrealistic.

Page 19, Lines 322-332 (Discussion): First, the morphological similarity of surface features between adenomas and cancers likely limited the effectiveness of short-term training. Second, because colorectal polyp diagnosis is inherently a visual task, providing a text-only training program to participants with predominantly verbal WM may have created a mismatch between instructional modality and task demands. Indeed, even within the Matched-E group, diagnostic accuracy for adenoma and intramucosal cancer was lower in the verbal-education subgroup than in the visuospatial-education subgroup (45.5% vs. 61.3%, P= 0.016; Supplementary Table 3). Thus, verbal instruction may not have been fully aligned with the ultimate goal of improving polyp diagnostic ability. In particular, the second reason suggests that direct application of this approach to clinical practice is challenging. It is not feasible in real-world training to fully segregate trainees into verbal or visual WM groups.

**Comment 3:**

Specification of Future Directions
The statement that “longer training may be necessary” is insufficiently specific. The Discussion should propose concrete strategies for overcoming the identified limitation, such as incorporating visually intensive instructional components or hybrid methods that integrate both visual and verbal modalities, tailored to WM profiles for tasks with high visual demands.

**Response to Comment 3:**

We are grateful for the clear guidance provided to improve the quality of our manuscript. As the reviewer correctly pointed out, simply extending the duration of training is insufficient. As noted in our responses to Comments 1 and 2, for tasks that fundamentally require the acquisition of visual diagnostic skills, providing prolonged verbal-only instruction to trainees with predominantly verbal WM is inefficient. For tasks such as polyp diagnosis, it may be appropriate to emphasize visual instruction for trainees with predominantly visual WM, whereas for those with predominantly verbal WM, a hybrid approach that combines visual training with verbal elements would be more practical.

Previous reports on short-term training for polyp differentiation using classification systems (Raghavendra M et al., GIE 2010; Ignjatovic A et al., GIE 2011) also employed visual and text-based teaching materials. These findings suggest that purely verbal or visual instruction is not efficient in practice. Thus, it would be more realistic for trainees and trainers to consider the predominant WM profile and apply a mixed, hybrid approach—placing greater emphasis on one modality but still incorporating the other—rather than relying exclusively on a single type of instruction.

In line with this suggestion, we have removed the statement “Thus, a longer training intervention may be necessary to observe the difference in distinction accuracy for neoplastic lesions between the Matched-E and Unmatched-E groups.”

Immediately following this section, we have added a new paragraph that discusses the possible reasons why diagnostic accuracy for neoplastic lesions such as adenomas and invasive cancers did not improve, and proposes more practical educational strategies to address this limitation.

Page 19, Line 332 to Page 20, Line 338 (Discussion): Reports on short-term training using classification systems have incorporated image-based and text-based materials [6,7], further suggesting that purely verbal or purely visual approaches are inefficient. For inherently visual diagnostic tasks such as polyp classification, extended verbal-only training is unlikely to be effective for individuals with verbal WM dominance. A more practical strategy would be to apply hybrid methods, focusing on visual education for visual WM-dominant trainees while combining verbal and visual instruction for verbal WM-dominant trainees.
